# Towards Process-Oriented Hospital Structures; Drivers behind the Development of Hospital Designs

**DOI:** 10.3390/ijerph20031993

**Published:** 2023-01-21

**Authors:** Jeroen D. H. van Wijngaarden, Anoek Braam, Martina Buljac-Samardžić, Carina G. J. M. Hilders

**Affiliations:** 1 Health Services Management & Organisation, Erasmus School of Health Policy and Management, Erasmus University Rotterdam, 3062 PA Rotterdam, The Netherlands; 2 Raad van Bestuur, Reinier de Graaf Gasthuis, 2625 AD Delft, The Netherlands

**Keywords:** hospital design, multi-disciplinary cooperation, integrated care, multi-morbidity

## Abstract

Hospitals have been encouraged to develop more process-oriented designs, structured around patient needs, to better deal with patients suffering from multi-morbidity. However, most hospitals still have traditional designs built around medical specialties. We aimed to understand how hospital designs are currently developing and what the important drivers are. We built a typology to categorize all Dutch general hospitals (61), and we interviewed hospital managers and staff. The inventory showed three types of hospital building blocks: units built around specific medical specialties, clusters housing different medical specialty units, and centers; multi-specialty entities provide the most suitable structure for a process-oriented approach. Only some Dutch hospitals (5) are mainly designed around centers. However, most hospitals are slowly developing towards hybrid designs. Competitive drivers are not important for stimulating these redesigns. Institutional pressures from within the health care sector and institutional ‘mimicking’ are the main drivers, but the specific path they take is dependent on their ‘heritage’. We found that hospital structures are more the result of incremental, path-dependent choices than ‘grand-designs’. Although the majority of the Dutch general hospitals still have a general design built around medical specialties, most hospitals are moving towards a more process-oriented design.

## 1. Introduction

Since the beginning of this century, hospitals have been encouraged to redesign and develop more process-oriented structures [1,2]. In a process-based organization design, the structure is built around patient needs, in which multi-disciplinary organizational departments (including multiple medical specialties) can each handle all the needs for specific patient groups, with few interdependencies between departments [2,3]. This seems especially important as the number of patients with multi-morbidity, especially multiple chronic diseases, is rising fast in many countries [4]. In general, such designs are expected to increase the quality of care and reduce costs, for which there is some evidence, and to improve patient-centered care [3,5]. However, in practice only a few hospitals have up till now opted for such a redesign; most hospitals still have a more traditional structure built around medical specialties [5].

In this study, we therefore aim to understand how hospital designs (organizational structures) are currently developing and what the drivers are behind these developments. We chose to perform this study in one country: the Netherlands. Although this does not allow us to study the influences of different systems, it does give us more opportunity to understand why different structures develop even when the contextual conditions are partly the same.

### 1.1. Theory

#### 1.1.1. Hospital Designs and Interdependencies

More traditional hospitals have a so-called functional design in which people with similar expertise or knowledge are grouped in organizational departments, mostly built around medical specialties, such as neurology [5]. However, as patients often rely on the expertise from different departments and specialties, interdependencies become difficult to manage. Both sequential (process) and reciprocal interdependencies play a role [6,7]. Sequential refers to the fact that during the course of their disease and treatment patients sequentially require help from different departments (as well as professionals and specialties), going, for example, first to the emergency department and then to the OR and ICU and then a medical ward (the output of one department (specialty) is the input for another; this is a (mostly) one-way street) [6,7]. Reciprocal dependencies relate to the fact that patients may require the help of different medical departments (professionals or specialties) during the same phase of their disease trajectory, because of multi-morbidity for example (both the output and the input of each specialist are interdependent: a two-way street) [6,7]. As the number of patients with multi-morbidity is rising fast, reciprocal interdependencies, especially between different medical specialties, are increasing in hospitals [4]. By creating so called clinical institutes, hospitals have tried to deal with these interdependencies [8]. Clinical institute designs organize services around patient conditions, such as cancer services and cardiothoracic care [8]. However, such a design often requires a major organizational restructuring. According to Vera and Kuntz [3], organizational restructuring is not the only path towards a more process-oriented structure; another option is to implement coordination mechanisms within existing structures (e.g., multi-disciplinary meetings and standardized care pathways).

#### 1.1.2. Drivers for Organizational Change

The structural choices that organizations make are at least partly based on their strategies. Although the adage ‘structure follows strategy’ has long been falsified and structures also develop through incremental decisions and changes, strategy and structure do influence each other [9,10]. Paauwe and Farndale [11] developed a framework to understand how organizational choices (about structures for example) are shaped by different drivers, namely institutional pressures, competitive drivers, and historically grown configurations [12,13]. First, organizational choices are subject to institutional pressures and the rules, norms, or values that are prevalent in the sector. In order to gain legitimacy and improve their chances of survival, organizations will conform to these “rules of the game” [11,12,13,14]. Second, competitive mechanisms influence organizational choices. In order to gain competitive advantage, organizations are driven to optimize effectiveness and efficiency [11,12,13]. Third, organizations are influenced by their own heritage. Historically grown configurations, based on past choices silicified in structures, roles, competences, and values, may form path-dependent patterns for future choices [11,12,13]. These three drivers shape the perceptions of those individuals with decision-making power, the dominant coalition, of the room they have to maneuver in and make specific choices. These drivers may therefore help us better understand why hospitals opt for specific designs.

## 2. Materials and Methods

### 2.1. Setting: The Dutch Hospital Sector

Dutch hospitals are mostly private, not-for-profit organizations, with a few exceptions. In 2019, there were 69 hospital organizations, with 116 hospital locations [15]. These 69 hospitals include eight university medical centers and 61 general hospitals. On average, a Dutch hospital organization has 450 beds [15]. In general hospitals, about 65% of the doctors are part of an independent medical specialist group (mostly based on specialty); 35% are employed and salaried (especially younger medical specialties such as geriatrics and intensivist and emergency physicians) [16]. Since 2015, these independent medical specialist groups have needed to negotiate their payment with the hospitals; before 2015, they negotiated independently with the insurers who act as health care purchasers in the Dutch system [17]. Therefore, in each hospital medical specialist groups now form a Medical Specialist Company together. Employed medical specialists are organized in many hospitals in an Association of Employed Medical Specialists. In many hospitals, specialists have chosen to unify their representation towards the board of directors by creating an Association of (all) Medical Specialists, which works in close cooperation with the hospital’s board of directors.

The Netherlands has a market-based system in which private, statutory insurers are responsible for the strategic purchasing of care for their clients. The insurers negotiate with hospitals over prices, quality, and volumes [17]. Purchasing health insurance from a private health insurer is obligatory for all residents in the Netherlands. Payment for hospitals is mostly based on a Dutch version of the diagnosis-related group approach. Hospitals are expected to compete on both quality and costs. The national government sets overall priorities for health care and monitors access, quality, and costs [17]. Every four years since 2012, the ministry of health has initiated an agreement with, among others, the Dutch Medical Association, the Dutch Association for Health Insurers, and the Dutch Association for hospitals about costs and quality. All of these agreements have put a cap on growth of expenses for specialist care. The 2019–2022 agreement states that in 2022 there should be a zero percent increase in expenses for specialist care [18].

### 2.2. Research Design

In this study, we used multiple qualitative research methods to study the development of hospital designs in the Netherlands. The study consisted of two phases. In the first phase of the study, we tried to obtain a general overview of how the designs of Dutch hospitals vary by studying the annual reports and organization charts of all the Dutch general hospitals. The second phase of the study was the most important for answering our research question; we interviewed hospital managers and staff of a selected number of these hospitals to understand what the drivers are behind different hospital designs.

#### 2.2.1. Phase 1

##### Data Collection

Between January and April 2019, five junior researchers visited the websites of all 61 Dutch general hospitals to acquire the annual reports and the organizational charts. If the organizational charts were not available online, they called the hospital to acquire them.

##### Data Analyses

The 61 general hospitals were divided between these five junior researchers, who each studied the relevant annual reports. Together, they presented all the organizational charts in one file and added relevant information from the annual reports. This file was analyzed by the first author to identify communalities and differences between hospitals in how the different medical specialties and professions were organized in departments to deal with sequential and reciprocal interdependencies. As the existing categorizations (e.g., traditional professional design versus clinical divisional and clinical institute directorates) did not capture the relevant variations we found, we decided to build a new categorization based on the data. The first author therefore developed a preliminary typology. This typology was discussed in several rounds with the other authors (the third author is a hospital director) until consensus was reached; this resulted in three basic types: unit, cluster, and center design. Based on this typology, the first and the second authors independently categorized each hospital. Some hospitals were difficult to categorize as the organizational charts were somewhat unclear because of the terminology used. As a consequence, the first and second authors categorized 12 hospitals differently: primarily into unit and cluster designs (there was only one center design, which was at that moment in transition, making the categorization difficult). Differences in opinion were discussed (using the charts and the annual reports) until consensus was reached.

#### 2.2.2. Phase 2

##### Data Collection

The categorization in the first phase was used in the second and most important phase to select hospitals. From each category, we selected three to six hospitals. In our selection, we also took the variations within categories into account: for example, hybrid structures and geographical spread. In order to understand the rationale behind the hospital designs, semi-structured interviews were conducted by the second author. We interviewed (at least) two respondents from each hospital; all the respondents were familiar with the choices made about the structure. For each hospital, we contacted the secretary of the board of directors and asked his advice about who to interview. From November 2020 to March 2021, a total of 26 interviews with representatives from 12 hospitals were conducted, including members of the board of directors, members of the medical advisory board, and medical managers (see Table 1).

We developed an interview guide, partly based on our findings in the first phase of the data collection, to deepen our understanding of the hospital structure and its development and partly on the framework of Paauwe, to understand the drivers behind the choices made. The respondents were first asked to describe the structural design of their hospital in their own words; this was followed by more detailed questions about the structure. Then, we asked how this structure had developed over time and the reasons why. We also asked about other developments/projects within the hospital that affected the structure. Subsequently, we discussed the different mechanisms from the Paauwe model; we asked about the influence of competition, stakeholders, population characteristics, and governmental regulations. We also asked about the influence of past strategic choices, existing structures, culture, and power distributions. Finally, we asked about the role of the dominant coalition in making choices related to the organizational structure. The first interviews were regarded as a pilot test (more than two interviews were conducted in this hospital). Only a few small changes were made to the guide based on this pilot test.

Due to the COVID-19 pandemic, all the interviews were held via the online platform Microsoft Teams or by telephone. All the interviews were in Dutch. The relevant citations were translated to English for this paper.

##### Data Analyses

The interviews were audio-recorded and transcribed verbatim and analyzed. We used a combination of deductive and inductive approaches to analyze the data. First, the interviews were deductively labelled by the second author using the different drivers identified by the model of Paauwe. Second, open coding was used by the first author to analyze each of the drivers and the relationship between the drivers. This process was followed by axial coding; the codes were clustered thematically to identify patterns in the developments of the hospitals. These patterns were checked by the second author in the data. Then, these patterns were discussed and adapted by the first and second authors until consensus was reached.

##### Ethics

The Ethics Review Board confirmed that our study was outside the scope of the Netherlands’ Medical Research Involving Human Subjects Act and that the rights and privacy of the study participants were sufficiently considered (METC-LDD-2019-Z19.0). All the respondents were asked for informed consent. All the data are stored and encrypted in a cloud server provided by our university and are only accessible by the authors of this paper.

## 3. Results

Our inventory shows that there are three types of basic building blocks for Dutch general hospitals; **units, clusters, and centers**. **Units** are built around specific medical specialties, such as internal medicine, pulmonary medicine, gastrointestinal liver disease, dermatology, urology, neurology, neurosurgery, etc. These units are responsible for organizing both inpatient and outpatient care and have their own (specialist) nursing staff. In some hospitals, these units are the main building blocks and have a lot of autonomy. **Clusters** are basically umbrellas under which different medical specialty units are housed. To allow the sharing of resources and stimulate cooperation, power is partly centralized from the unit level to the cluster level, although units still have a lot of autonomy. **Centers** are multi-specialty entities. In contrast to clusters, centers do not have separate specialty units within. Centers are often built around patient conditions, such as those for oncology and those for the elderly, the heart, etc., but they can also be based on care type, such as acute care, chronic care, and elective care. From our interviews, we learned that the choice to organize a center around a type of care relates to scale, as organizing all care around patient conditions would result in ‘too many’ (small) centers. The cluster design (37 of 61) is the most common in the Netherlands, followed by the unit design (19 of 61). Only a few hospitals (5 of 61) are designed around centers, although this design may be the best suited to introduce a process-oriented organization. However, there are hybrid forms, such as different hospitals (16) with primarily a unit or a cluster design but which also have one or a few centers, often focused on oncology, mother and child care, and/or heart–lung care. Moreover, within all hospitals coordination mechanisms are (being) introduced so that they can become more process-oriented. For example, lean principles or value-based health care (VBHC) principles are in many hospitals used to build patient care pathways and introduce multi-disciplinary meetings between specialties. However, the scale and tempo in which these coordination mechanisms are introduced differs a lot between hospitals. We also see some hospitals that are introducing a matrix-like structure (in line with VBHC principles), in which the management of capacities (beds, OR, etc.) is separated from the management of patient trajectories.

During the interviews, we asked the respondents about the structural design of their hospital, how it developed, and what the main drivers were for the choices they made. Based on the model of Paauwe, we distinguish between competitive drivers, institutional pressures, organizational heritage, and the role of the dominant coalition.

### 3.1. Competitive Drivers

From the interviews, we learned that outperforming competitors and growth is not a driver for redesigning Dutch hospitals towards a more process-oriented structure. Because of the need for cost containment in the Dutch health system, the insurers, together with the Dutch government, have put a cap on growth. The hospitals are only allowed a growth in production of a few percent each year and budgets remain tight. As a consequence, in the Netherlands the smaller general hospitals in particular are and have been struggling for survival. That is why in recent years there have been many hospital mergers: 27 between 2008 and 2018 [19]. Although these mergers required reconstruction, most chose not to change the fundamental design (unit or cluster), as the integration would have taken up all of their energy. Currently, many hospitals are still dealing with the aftermath of these mergers and are therefore not willing to undertake major revisions.

*“Much efforts have been spend to integrate speciality groups (e.g., groups of similar medical specialists (for example neurologists) from the different hospitals in the merger), mostly that has succeeded. But in some places you still see the remnants, which make you think they haven’t really fallen into each other’s arms yet, maybe on paper, but not in their culture, in the way they work nor in their views. So, within the different hospitals that have merged even specialists from the same discipline are not lined up yet”.*

At the same time, a growing demand for care, together with the need for cost containment, also stimulates hospitals to think about more efficient and effective ways of organizing care. Currently, most Dutch general hospitals provide similar services. To increase efficiency and quality, insurers and the Dutch government are stimulating hospitals now to specialize more through, for example, selective contracting. As a result, however, many Dutch hospitals are not competing but increasingly working together to divide care delivery between them.

*“What is interesting to mention is that I notice there is much mutual consultation between hospitals. I notice that we have many talks with the hospitals nearby on board level…do we need to make choices together about who does what? We concluded there are forms of basic care we all need to deliver. But some specialist care we can devide….so how can we improve cooperation, in which each of us is not doing everything (perform all treatments)”.*

### 3.2. Institutional Pressures

There seems to be a strong set of shared values within the Dutch hospital sector. All the respondents mentioned that they shared the ambition to work towards a more process-oriented structure and also the ambition to work more in regional networks with other care providers. However, they struggle with how to organize this. Different respondents referred to the Karolinska hospital in Sweden, which is one of the first hospitals which was completely restructured towards a clinical institute design, as an example or inspiration:

*“We started work-conferences with our specialists, that’s where we lay the foundation for thinking in terms of multi-disciplinary teams. It is also when we visited Karolinska”.*

Dutch hospitals also often look to each other for inspiration on how to work towards these ambitions:

*“We looked at the outside world, how do others do this,…then you see slowly the development towards more care oriented, network oriented and matrix like structures”.*

On the one hand, the sector is a strong reference point that inspires; on the other hand, existing structures and regulations within the sector are also seen as inhibiters. One of the respondents saw the current medical education of physicians as an important inhibiter because the students are mostly trained in the silo of a specific specialism and do not think in terms of multi-disciplinary care pathways:

*“As long as we educate our medical students in the traditional specialist silo’s…this mono-disciplinary focus will remain. I think it requires a few generations of medical students, to slowly develop towards care pathways”.*

### 3.3. Heritage

Different respondents stated that many characteristics of their current design were not so much driven by strategic choice but were the result of small pragmatic consecutive changes. Although the main design for a unit or cluster structure was a fundamental choice in the past (mostly more than 10 years ago), over time pragmatic choices were made to deal with new circumstances. Past choices and existing structures often guided future choices. One respondent gave an example of how the choice was made for the number of directors and therefore the number of departments:

*“and again that is something that just came about, before we had four managers and we went back to three, I think it just depended on what talent is available and what works. You do not want too many directors, but also not too little”.*

Some respondents mentioned how sometimes pragmatic choices resulted in very illogical structures:

*“When I came to work here, there where some, so to say, ridiculous combinations…What was the person thinking that put these units together, what is the logic behind this? And when I started asking, it was like…yea, that was all the one in charge could handle at the time, so this part needed to go and we just put it there”.*

*“they had this fun saying, about things that happened in the past. They said: this is hysterically grown”.*

However, at the same time, these small consecutive steps can also be driven by strategic choices. In particular, when it comes to creating a more process-oriented structure, different respondents stated that it was mostly about seizing opportunities and gradual change, sometimes even covert actions.

*“well we’ve been working on this for the past years, but more or less in an organic manner. Somebody retired, who was in charge of 3 units, and we took the opportunity to redistribute these units in a more sensible way…We are working towards what we call ‘Patient Responsible Units’…clustered around themes (for example Chronic Care)…And we try to slowly build the portfolio’s of our managers around those themes. So each manager will finally have two themes. And hey presto..surprise suddenly it is there”.*

Most hospitals shy away from sudden major reforms and prefer a more incremental approach. Lack of stability was mentioned several times as a reason, because of past mergers (as discussed before) or financial instability:

*“a couple of years ago we talked about the ‘Karolinska model’, you’ve probably heard of it…but these last years we had to cut back 30 million (euro’s) without reduction in productivity. When you want to do something like that (restructuring) you need to let go of normal budgeting procedures and your organizational design. You need to change these, mess it all up, which is quite complicated. That is not something you can do when you’re sailing close to the wind. So, we pushed that forward, although we are taking small steps”.*

Another reason the respondents often referred to was that redesigns may harm the interests of doctors or, more specifically, some specialists, as a new structure will divide subspecialties between departments. That is why centers are often built around patient groups that do not require the main specialisms involved (especially dominant specialisms) to be split up in different centers and where there is already a tradition of intensive multi-disciplinary cooperation, such as oncology.

*“…fear of losing influence and power. At the moment, specialisms have a strong mandate. So they have little to gain by doing things differently, So they resist; well some do…When your budget is divided between two Result Responsible Units or themes, then others control your income. And then you need to involve others in decision making”.*

### 3.4. Dominant Coalition

All the respondents referred to the board of directors together with the medical representatives as the dominant decision makers in the hospital. The hospitals that did make the decision to fundamentally redesign towards a more process-oriented structure all seemed to have a stable, visionary board of directors and strong, supportive medical representation. It seems that it takes a decisive and tenacious dominant coalition to successfully initiate and implement a redesign.

*“So how did it all come about (the redesign towards a hospital build around centers), I think our director (a former medical specialist) was an important driver…she always said that it is important that medical specialists take the lead together with general managers in a hospital…In every hospital there is not a single line structure, but there is the hierarchical line and next to this the medical specialists with their own mandate, and this always creates a hassle…So I was very glad when…(name director) said, that we need to put specialists more in the lead”.*

A number of respondents mentioned how their hospital was not ready for a major redesign towards a process-oriented structure, although they wanted to, because there was no stable board of directors:

*“I think in that context, where we came from, there was momentum, in which we all thought we need to do something now with that philosophy (process-oriented), otherwise we will be ten years on. But we had a change in the board of directors and the interim director didn’t want to turn things completely on its head. So, this was the most feasible solution”.*

Additionally, other respondents mentioned that the representation of their doctors was fragmented and therefore somewhat rudderless, which slowed down or inhibited change.

*“…when I was cluster-manager, when I wanted something I needed to visit all these groups (specialisms) and they all needed to agree. It was all very fragmented really and the medical staff was also somewhat rudderless, because they lacked a well-established structure for representation. So, he (the new director) said from the beginning, I want to govern together with the medical staff, but then I need one representative”.*

From the interviews, we learned that most of our hospitals are trying to work towards a more process-oriented approach, but mostly through incremental change and not through redesigning the main structure in one go. At the same time, our respondents in those hospitals that had redesigned their structure, mentioned they were still struggling to really change their way of working. Although the structural conditions have changed, underneath the old patterns still exist of specialisms that are used to working together and others that are hesitant to do so. Consequently, multi-disciplinary cooperation and patient pathways still need to be improved or even introduced.

*“Preferable we would like to change towards RRU’s based on care-pathways…, That works fine for mother-child and for an oncology center, but you also want to take the perspective of the older patient, so organize this for geriatrics and maybe for trauma. But we notice that this is really complicated”.*

## 4. Discussion

Our inventory of all the Dutch general hospitals shows that their structure can be categorized based on three types of basic building blocks: units, clusters, and centers. This categorization shows some similarities but also important differences with the existing categorizations [8]. It seems that Dutch general hospitals do not use a traditional professional design (anymore) as there is no organizational division between medical and nursing staff (ibid). In each design, in all types of medical departments, both nurses and doctors are housed. Hospitals primarily based on units clearly resemble a clinical divisional design, as these units are mostly built around single medical specialties such as neurology [8]. They basically group services around ‘*the way medicine is organised’* (ibid, 2). Hospitals that use clusters as an important building block cannot be easily related to existing categorizations, and this is the most prevalent design in the Netherlands. In this design, different specialisms are ‘clustered’ that have similar work processes and patient trajectories and therefore require similar facilities and support structures. This design allows them to better deal with sequential interdependencies. However, within these clusters traditional units often still play a dominant part and the coordination between them is not guaranteed; therefore, sequential interdependencies between specialties are less dealt with. Hospitals built around centers mainly resemble clinical institute designs as services and are often organized around patient conditions, such services for oncology and obesity [8]. However, a center can also be organized around care types such as acute care, chronic care, and elective care. Typical for all centers is their multi-specialties approach, in which traditional units are no longer relevant. Different hospitals use a combination of design logics to organize their centers. This seems to be related to scale because organizing all care around patient conditions would result in ‘too many’ (small) centers. The research suggests that such centers better allow for a process-oriented approach, dealing with both sequential and reciprocal interdependencies and leading to better outcomes [5]. However, in these hospitals the underlying forms of coordination often still need to be implemented to be able to reap these potential benefits.

It is important to notice that most Dutch hospitals slowly develop towards hybrid designs by using combinations of building blocks and design logics. As already mentioned, in hospitals with clusters units are still relevant, but in both cluster and unit hospitals, we also increasingly see the introduction of centers, especially around medical conditions that require intensive multi-disciplinary cooperation, such as oncology. At the same time, most hospitals are now introducing coordination mechanisms between and within existing building blocks (based on lean or value-based health care principles) to better deal with both sequential and reciprocal interdependencies. Some unit hospitals claim that their small size already allows for easy coordination, without the need to redesign their basic structure. This seems in line with the findings from a review on process redesign methods in which forty-one percent of the studies found success in ‘*changing employee practices to improve care processes, without additional resources or structural change’* [20]. Our findings show that most Dutch general hospitals opt for incremental change towards a more process-oriented design, instead of radical redesign.

We used the model of Paauwe to understand how these choices are shaped by institutional pressures, competitive drivers, and historically grown configurations and by the dominant coalition (of decision makers) [11,12,13]. This model was very helpful in identifying and categorizing underlying mechanisms. Our study shows that within the Dutch health care system, competitive drivers are not important for redesigning Dutch hospitals towards a more process-oriented structure, while authors such as Porter strongly relate this development to the creation of competitive advantage [21]. The reason is that although there is market competition, the Dutch government has put a cap on the growth of the expenditure of hospital care, while demand is still growing. As a result, hospitals are increasingly cooperating instead of competing to deal with rising demands (see also [22]). It seems that cost containment is more of a driver behind the restructuring of Dutch hospitals than competition. Insurers are stimulating hospitals to focus more on cost containment, but they still leave it to the hospitals to choose the structural changes they want to make, be they the introduction of coordination mechanisms, a structural redesign, or a combination of both. Normative pressures from within the health care sector and institutional ‘mimicking’ [23] are especially relevant for pushing the agenda. There seems to be a shared ideal in the Dutch care sector that hospitals should be organized in a more process-oriented manner and more around the patient’s needs in order to deliver better quality. There is much consensus on where to go, but not on how to get there. Each hospital follows its own course, which is very much dependent on historically grown configurations of past decisions, existing structures, and power distribution (especially regarding the doctors). The first steps are therefore often taken in redesigning processes in which the doctors are already working intensively together, such as oncology. In other words, most hospital structures seem to be more the result of incremental, path-dependent choices than ‘grand-designs’. Hospitals that do choose radical redesign seem to have a number of characteristics in common. They all have a stable, visionary board of directors and strong supportive medical representation on a strategic level (a strong dominant coalition). Only then are boards of directors able to go against the vested interests of (some) medical specialties, which will be affected by the redesign. These findings seem to be in line with different studies that show how important the support of doctors is for successful changes in hospitals [20,24].

Other studies have also shown that efforts to stimulate multi-disciplinary cooperation are not always supported by doctors. Discussions about professional domains and autonomy are often found to be the cause [24,25]. In particular, when professional domains (partly) overlap, multi-disciplinary cooperation can result in turf wars [24,25]. For example, vascular surgeons and intervention radiologists provide alternative treatments for some of the same vascular problems. However, in one of the hospitals they told us that these specialists rarely cooperate and some even refuse to cooperate. Moreover, specialists can be hesitant to give up the large amount of autonomy they have in more traditional hospital structures. However, it also seems to depend on how much awareness there is of interdependency. For some patient conditions, the interdependency between different specialisms is more obvious and frequent than for others. An orthopedic surgeon can treat many of his patients without the aid of other medical specialists (except for support specialists, such as anesthesiologists). For these specialists, sequential (process) interdependencies are more important than reciprocal interdependencies. They will focus more on the development of care pathways within existing structures than on redesigning the organization towards multi-disciplinary centers. However, oncologists are for the treatment of most of their patients dependent on other specialists. Both sequential and reciprocal interdependencies are important for them. They are therefore probably more likely to support the development of centers.

This study has a number of limitations. First of all, organizational diagrams can be an outdated or idealized representation of an organizational structure. They also do not show how coordination and steering actually take place. However, they do give a general idea of the structure and the choices that are made, and they help to identify the most important differences and communalities between hospitals, which was important for this study. Second, we only approached 12 hospitals of the 61 hospitals for phase 2; therefore, there may be a selection bias. We also expected a more or less even distribution between the three hospital types we identified based on our sample selection. However, in practice more hospitals were of the unit type, showing that it is difficult to correctly categorize hospital structures based on only organizational charts and annual reports. At the same time, the findings from our interviews seem to support our typology; they also confirmed that there are different hybrid approaches, and they confirmed importance of coordination mechanisms to develop more process-oriented structures. Third, we decided not to perform a member check because our conclusions and analyses were not related to specific hospitals but were based on a comparison between hospitals. Still, a member check could have given us additional information which may have been relevant for validating our findings. Fourth, we only interviewed two respondents for most of the hospitals that we sampled. This could have introduced a bias in the information that we obtained. However, we did try to speak to those representatives that could give us the best overview of the choices made and the steps taken in (re) structuring these hospitals. Finally, we only researched hospitals in the Dutch health care system, which has its specific characteristics, such as little competition between hospitals. This probably has an effect on the generalizability of our findings. In more competitive systems, market forces will probably play a stronger role. We do think that in most systems hospitals are complex organizations to change and are strongly dependent on the cooperation of the doctors. Therefore, we expect that restructuring will often be more the result of incremental, path-dependent changes than the product of ‘radical redesigns’.

## 5. Conclusions

Hospitals increasingly have to take care of patients that suffer from multi-morbidity and often multiple chronic diseases. While these patients need help from different specialties, the research suggests that hospitals are still mostly organized in silos around specific medical specialties, which may inhibit multi-disciplinary cooperation. However, our study seems to show a more nuanced picture. Most Dutch hospitals are moving towards a more process-oriented design, not through radical redesign, but by introducing coordination mechanisms and the development of multi-specialty centers. Institutional pressures from within the health care sector and institutional ‘mimicking’ are the main drivers for these changes, but the specific path they take is dependent on their ‘heritage’. Still, these changes especially concern specialisms in which the majority of the patients suffer from multi-morbidities. Making sure other specialisms also start cooperating may require strong medical leadership at a strategic level.

## Figures and Tables

**Table 1:** Respondents.

Hospital Type	Hospital	Function Respondents
Unit design	A	Chairman of the board of directors
	A	Urologist and project manager strategy
	B	Chairman of the medical staff association
	B	Pediatrician and secretary medical specialist company
	C	Secretary of the board of directors
	C	Secretary medical staff association
	D	Secretary of the board of directors
	D	Secretary medical staff association
	E	Secretary of the board of directors
	E	Chairman medical specialist company
	F	Chairman of the board of directors
	F	Manager of a staff department
	F	Gynaecologist and chairman medical coordinators
Cluster design	G	Secretary of the board of directors
	G	Secretary medical staff association
	H	Secretary of the board of directors
	H	Manager human resources
	I	Secretary of the board of directors
	I	Manager strategy and sales
Center design	J	Secretary of the board of directors
	J	Chief medical department
	K	Secretary of the board of directors
	K	Business manager of a medical department
	K	Business manager of a medical department
	L	Secretary of the board of directors
	L	Business manager of a medical department

## Data Availability

Data are partly available on request as the interview transcripts contain much personal information that is difficult to anonymize.
